# Focal ^123^I-FP-CIT SPECT Abnormality in Midbrain Vascular Parkinsonism

**DOI:** 10.1155/2015/642764

**Published:** 2015-10-15

**Authors:** Paolo Solla, Antonino Cannas, Roberta Arca, Davide Fonti, Gianni Orofino, Francesco Marrosu

**Affiliations:** Movement Disorders Center, Department of Neurology, Institute of Neurology, University of Cagliari, Monserrato, 09042 Cagliari, Italy

## Abstract

Cerebrovascular diseases are considered among possible causes of acute/subacute parkinsonism, representing up to 22% of secondary movement disorders. In cases of suspected vascular parkinsonism (VP), dopamine transporter SPECT has been highly recommended to exclude nigrostriatal dopaminergic degeneration. We report the case of a hemiparkinsonism related to a left midbrain infarct with focal lateralized putaminal abnormalities at ^123^I-FP-CIT SPECT imaging. The asymmetric uptake at dopamine transporter SPECT was different to findings commonly observed in typical PD pattern, because the ipsilateral striatum, in opposite to idiopathic PD, showed normal tracer binding. However, this selective parkinsonism after infarction of the midbrain was responsive to levodopa. In conclusion, we retain that there is a need of more functional imaging studies in VP addressed to a more consistent classification of its different clinical forms and to a better understanding of the adequate pharmacological management.

## 1. Introduction

Parkinsonism with acute/subacute onset is uncommon and other conditions, different from a neurodegenerative process, should be considered in differential diagnosis. In this context, cerebrovascular diseases are considered among these possible causes of parkinsonism, representing up to 22% of secondary movement disorders [1]. In this regard, mesencephalic infarcts have been related to parkinsonism [2], often with rapid onset, and dopamine transporter SPECT has been highly recommended to confirm or exclude nigrostriatal dopaminergic degeneration [3].

We report the case of a hemiparkinsonism related to a left midbrain infarct with peculiar asymmetrical putaminal abnormalities at ^123^I-FP-CIT SPECT imaging.

## 2. Clinical Description

**Figure 1:**
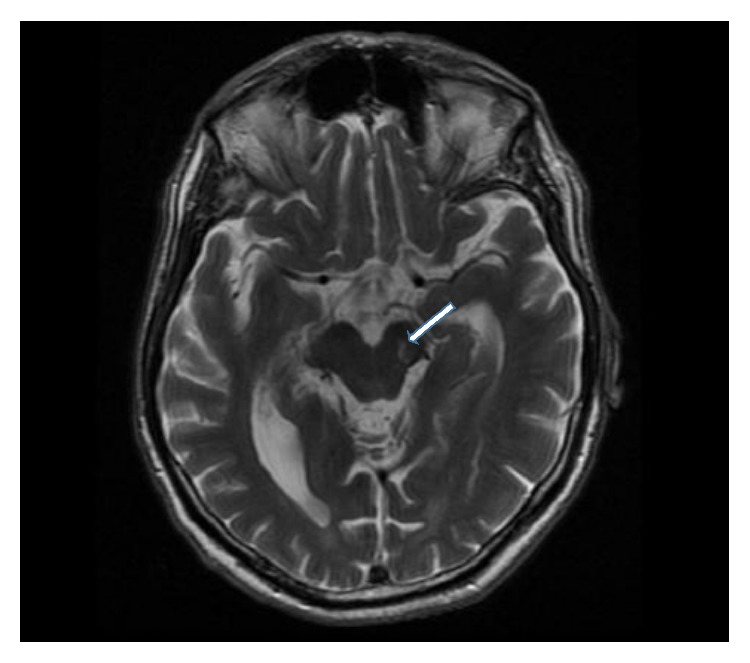
Cranial MRI revealed a lacunar infarction localized in the left midbrain (arrow) appearing hyperintense in T2 sections.

A 69-year-old male patient come to our observation suffering from motor slowness and rigidity at right limbs which showed an acute onset. His medical history revealed diabetes and a previous psychiatric history of depression. Neurological examination showed severe bradykinesia and rigidity in his right hemibody, with inconstant mild rest tremor. The patient was mildly depressed, without significant cognitive impairment or other important nonmotor features. A brain MRI was performed two months before and revealed a lacunar infarct in the substantia nigra (Figure 1) with associated features of cerebral small vessel disease with multiple subcortical lacunar infarcts.

**Figure 2:**
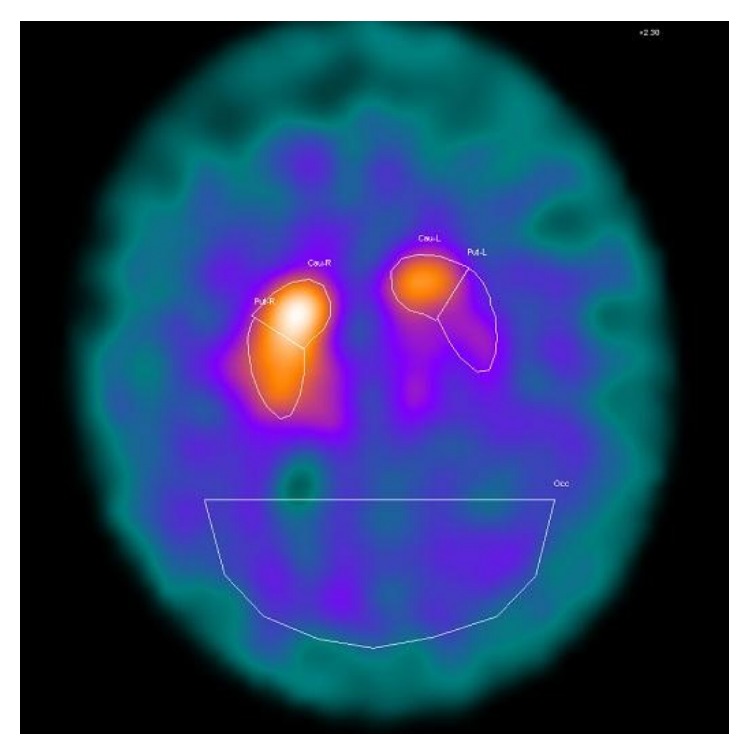
^123^I-FP SPECT showing a reduced tracer binding in the left striatum, with a more marked reduction in the putamen.

^123^I-FP-CIT SPECT showed an absent ligand binding in the left putamen and a less marked reduction in the homolateral caudate (Figure 2). Levodopa (400 mg/daily) was prescribed with moderate response. UPDRS motor score performed before levodopa introduction was equal to 27, while after 3 months it was 18. At the last follow-up visit, after 2 years, UPDRS motor score was not increased.

## 3. Discussion

The term vascular parkinsonism (VP), firstly described by Critchley in 1929 [4], is often used to indicate a progressive and bilateral parkinsonism related to cerebral small vessel disease with multiple subcortical lacunar infarcts and cited as “lower body” parkinsonism because the symptoms and signs predominate in the legs [5]. The concept of VP is a poorly defined entity, probably because of its heterogeneity (white matter lesions, basal ganglia involvement, and rarely substantia nigra lesions; diffuse ischemic changes versus discrete infarcts). Thus, VP cannot be considered as a unique entity that can be clearly differentiated from Parkinson's disease [3, 6].

Previous observations have described the occurrence of hemiparkinsonism following strategic infarcts affecting the basal ganglia, following contralateral lenticular [7, 8] or midbrain infarcts [2, 7, 9]. Moreover, although several findings have suggested that dopamine transporter SPECT might be normal in most patients with VP [10], conclusive data are still controversial [11].

Interestingly, a previous case with an isolated ischemic focal lesion located in the left cerebral peduncle and a homolateral SPECT abnormality has been described, although, different to our patient, dopaminergic treatment did not exert a definite positive response [12].

In our patient, the asymmetric uptake at dopamine transporter SPECT was different to findings commonly observed in typical PD pattern, because the ipsilateral striatum, in opposite to idiopathic PD, showed normal tracer binding. Moreover, the contralateral reduction in dopamine transporter uptake in the striatum at SPECT imaging was ipsilateral to this midbrain ischemic lesion, suggesting a probable involvement of the substantia nigra pars compacta.

In this regard, the selective vascular damage in the midbrain might have played a similar action observed in neurolesional animal models used in experimental settings.

This selective VP due to a midbrain lesion secondary was responsive to levodopa. This consideration is further supported by the evidence that also other conditions such as perivascular spaces in the midbrain can cause hemiparkinsonism and dopamine transporter SPECT abnormalities with documented dopamine response [13].

## 4. Conclusion

Correlation between VP and CIT SPECT is not a resolved issue. According to other previous study [10, 13], our findings confirmed that also VP patients can present with a positive response to dopaminergic treatment and this characteristic should not be considered as a peculiarity. In this context, however, the possibility of a coincidental finding with an initial degenerative parkinsonism, although less likely due to the lack of the motor impairments progression after two years, cannot be totally excluded. This case further suggests that there is a need of more functional imaging studies in VP with a more consistent classification of its different clinical forms. Such studies should explore more systematically the correlation of these forms with possible reduced uptake at dopamine transporter SPECT and its response to dopaminergic treatment.
